# Using Twitter to Measure Public Discussion of Diseases: A Case Study

**DOI:** 10.2196/publichealth.3953

**Published:** 2015-06-26

**Authors:** Christopher Weeg, H Andrew Schwartz, Shawndra Hill, Raina M Merchant, Catalina Arango, Lyle Ungar

**Affiliations:** ^1^ Positive Psychology Center Department of Psychology University of Pennsylvania Philadelphia, PA United States; ^2^ Department of Computer and Information Science University of Pennsylvania Philadelphia, PA United States; ^3^ Operations and Information Management Wharton School University of Pennsylvania Philadelphia, PA United States; ^4^ Social Media and Health Innovation Lab Department of Emergency Medicine University of Pennsylvania Philadelphia, PA United States; ^5^ Wharton School University of Pennsylvania Philadelphia, PA United States

**Keywords:** bias, data mining, demographics, disease, epidemiology, prevalence, public health, social media

## Abstract

**Background:**

Twitter is increasingly used to estimate disease prevalence, but such measurements can be biased, due to both biased sampling and inherent ambiguity of natural language.

**Objective:**

We characterized the extent of these biases and how they vary with disease.

**Methods:**

We correlated self-reported prevalence rates for 22 diseases from Experian’s Simmons National Consumer Study (n=12,305) with the number of times these diseases were mentioned on Twitter during the same period (2012). We also identified and corrected for two types of bias present in Twitter data: (1) demographic variance between US Twitter users and the general US population; and (2) natural language ambiguity, which creates the possibility that mention of a disease name may not actually refer to the disease (eg, “heart attack” on Twitter often does not refer to myocardial infarction). We measured the correlation between disease prevalence and Twitter disease mentions both with and without bias correction. This allowed us to quantify each disease’s overrepresentation or underrepresentation on Twitter, relative to its prevalence.

**Results:**

Our sample included 80,680,449 tweets. Adjusting disease prevalence to correct for Twitter demographics more than doubles the correlation between Twitter disease mentions and disease prevalence in the general population (from .113 to .258, *P* <.001). In addition, diseases varied widely in how often mentions of their names on Twitter actually referred to the diseases, from 14.89% (3827/25,704) of instances (for stroke) to 99.92% (5044/5048) of instances (for arthritis). Applying ambiguity correction to our Twitter corpus achieves a correlation between disease mentions and prevalence of .208 ( *P* <.001). Simultaneously applying correction for both demographics and ambiguity more than triples the baseline correlation to .366 ( *P* <.001). Compared with prevalence rates, *cancer* appeared most overrepresented in Twitter, whereas *high cholesterol* appeared most underrepresented.

**Conclusions:**

Twitter is a potentially useful tool to measure public interest in and concerns about different diseases, but when comparing diseases, improvements can be made by adjusting for population demographics and word ambiguity.

## Introduction

### Background

Word-use patterns in Twitter, Facebook, newsgroups, and Google queries have been used to investigate a wide array of health concerns. Twitter is perhaps the most popular online data source for such studies, due in part to its relative accessibility. It has been used to monitor health issues including influenza [1,2], cholera [3], H1N1 [4-6], postpartum depression [7], concussion [8], epilepsy [9], migraine [10], cancer screening [11], antibiotic use [12], medical practitioner errors [13], dental pain [14], and attitudes about vaccination [15].

Such research has demonstrated the utility of mining social media for public health applications despite potential methodological challenges, including the following: (1) Twitter users form a biased sample of the population [16-18], and (2) their word usage within tweets can be highly ambiguous. For example, focusing just on the medical domain, “stroke” has many nonmedical uses (“stroke of genius” or “back stroke *”* ); most mentions of “heart attack” are metaphorical, not literal (just had a heart attack and died the power went out while I was in the shower); and although doctors associate “MI” with myocardial infarction, on Twitter it refers more often to the state of Michigan.

### Study Objectives

This paper quantifies, and provides a framework for partially correcting, the error arising when using sources such as Twitter as a proxy for measuring disease prevalence. We investigate the relationship between the frequency of disease mentions on Twitter in the United States and the prevalence of the same diseases in the US population. Understanding this relationship could be useful for a variety of applications, including health care messaging and disease surveillance. We use Twitter as the venue for measuring discussion largely because it has already received substantial attention as an inexpensive proxy for tracking disease prevalence [19,20].

Our key contribution is to demonstrate that it is possible to better align Twitter disease-mention statistics with actual disease-prevalence statistics by correcting for ambiguous medical language on Twitter, and by correcting for the difference between the demographics of Twitter users and the general US population. We observe that a slight correlation exists between general population disease-prevalence statistics (sourced from existing survey data) and the number of times each disease is mentioned on Twitter (according to our own counts). We find that we can significantly increase this correlation (1) by restricting the disease-prevalence population specifically to Twitter users (ie, by correlating with existing prevalence data focused specifically on that group), and (2) by adjusting our disease-mention counts to correct for word-sense ambiguity.

## Methods

### Overview

We first identified a list of diseases; then for each disease, we constructed a list of terms that refer to it (ie, a disease-specific lexicon). We also collected a large number of tweets and compiled them into a tweets corpus. Next, we retrieved a random sample of tweets from our corpus that contained any of our disease terms. We then determined the relative frequencies (percentage) of medical uses of the disease terms (ie, valid positives) versus nonmedical uses (ie, false positives due to ambiguity), using human annotation on the random sample. This allowed us to compute corrected counts of the number of tweets in the corpus that mention each disease (we call this a disease’s “validated tweet count,” whereas an uncorrected count is termed a “raw tweet count”).

We correlated the corrected disease-mention frequencies with the US disease-prevalence statistics from the Simmons National Consumer Study [21]. The resulting correlation serves as a measure of the relationship between the quantity of disease mentions in the corpus, and the quantity of disease cases in the US population (for either the general population or the Twitter-using population). Comparing the correlations with and without correction demonstrates the size of our corrections.

### Data Collection

#### Selection of Diseases

We used the following criteria for selecting diseases for this research: (1) diseases that could be paired with both US population prevalence data and Twitter-use data; and (2) diseases deemed by previous literature to be most impactful for the health care community. Each criterion is satisfied by a different dataset.

The first dataset comes from Experian, a global information services company. Experian also conducts consumer surveys on a variety of topics, including health care. For this study, we used data from Experian’s Simmons National Consumer Study and focused on survey questions pertaining to general demographics, health status, and social media use.

Results from the various Experian surveys are combined into a database and released both quarterly and annually. Experian conducts poststratification on its survey data to create demographically representative estimates for its measured variables. We queried this database to obtain a dataset for the year 2012 that crosstabulates disease prevalence for all available diseases (n=52) with both general demographics and Twitter use. For the estimated English-speaking or Spanish-speaking US adult population (n=230,124,220), we were able to find the estimated number of individuals who suffer from a disease (eg, backache, n=42 million), and the subset of those disease sufferers who use Twitter (in the case of backache, n=2.6 million). This dataset, then, provides us with parallel sets of disease-prevalence statistics for the general US population and for US Twitter users.

The second dataset is from a RAND study designed to broadly measure the quality of health care delivery in the United States [22]. Through reviews of the literature and of national health care data, and through consultation with panels of medical experts, 46 “clinical areas” were identified in this report that represent the leading causes of illness, death, and health care utilization in the United States.

The list of 24 diseases (see Multimedia Appendix 1) used in this study is composed of the overlap between the diseases represented in the Experian dataset (n=52) and in the RAND study (n=46). This overlap may be explicit (eg, “asthma” appears on both lists) or implicit (eg, two separate Experian entries, “stomach ulcers” and “acid reflux disease/GERD,” are both suggested by the single RAND entry “peptic ulcer disease and dyspepsia”). The focus in this task was not pinpointing exact matches between the two lists, but rather finding areas of general agreement between them, to identify high-impact diseases from the Experian dataset.

#### Compilation of Disease Terms

For each disease on our list, we constructed a lexicon of disease terms that are used to refer to that disease. For example, the lexicon for *diabetes* used in this study contains three disease terms, namely, “diabetes,” “diabete,” “niddm.” All lexica in this study are derived from terms found in Consumer Health Vocabulary (CHV) [23], an online open source thesaurus that associates medical concepts (including diseases, medical procedures, drugs, anatomy, etc) with a mix of colloquial and technical terms. At the time of this study, the CHV contained 158,519 entries, covering 57,819 unique (but often closely related) concepts. Each entry collects (along with other data) at least three term elements: (1) a CHV term, (2) a descriptive phrase, and (3) a related term from a medical vocabulary called the “Unified Medical Language System (UMLS).” A CHV term can have multiple entries in the thesaurus, thereby associating the CHV term with any number of descriptive phrases or UMLS terms. Each CHV term can then be seen as a key-value pair, where the CHV term is the key and a network of associated terms (consisting of descriptive phrases and UMLS terms) is the value.

For each of the 24 diseases included in the study, we processed the CHV to retrieve an entire key-value network of associated terms if *any one* of the terms (in the key or the value) seemed to refer to the target disease. Multiple networks could be (and often were) collected for any disease. Together, these results constituted a disease’s list of candidate disease terms (these were then vetted, according to the process described in the “Vetting Disease-Term Candidates” section). A term was judged to be a potential reference to a target disease (thereby triggering the retrieval of all associated terms) primarily if it contained a search string derived from the target disease’s name (including both abbreviated and spelled-out forms). For example, “attention deficit” is a search string for *attention deficit disorder/attention deficit hyperactivity disorder (ADD/ADHD)* ; “heart disease” is a search string for *heart disease* ; and “GERD” is a search string for *acid reflux disease/GERD*. Also included among the search strings were some common disease synonyms, such as “zit” for *acne* and “tumor” for *cancer*. The number of search strings varied for each disease, ranging from 1 to 7.

#### Tweet Text Corpus

The tweets used in our analysis were taken from a random sample of 1% of all available tweets in 2012, as collected through the Twitter “1% random public stream” application programming interface (API) [24]. To align our data more closely with the American and mostly English-speaking Experian Simmons sample, we filtered our Twitter corpus to keep only English tweets originating in the United States. To filter for English, we only considered tweets with at least 50% of their words found in the Hunspell English dictionary [25]. Tweets were then further restricted to the United States by finding tweets with “United States” or nonambiguous US cities in their location field (city names were taken from [26]). For example, “Chicago” would match the United States, whereas “London,” even though there is a London in Texas, would not. This resulted in a corpus of 80,680,449 tweets.

### Vetting Disease-Term Candidates

#### Grammatical

In this research, we focused on finding tweets that specifically *name* our target diseases. Broadening this focus to include related concepts, such as symptoms and treatments, was desirable but was not possible for the scope of this paper. Because of our focus on terms that name diseases (as opposed to terms that describe or suggest them), we dropped all candidate disease terms that were not nouns (eg, adjectives such as “depressed” or “arthritic”). We then manually expanded the list, adding plural forms where grammatically appropriate.

#### Medical

We mined the CHV using a keyword search strategy inclined toward inclusiveness. For example, a search on “acne” retrieved terms for medical concepts that might be at best tenuously related to *acne*. One of these concepts was *acne rosacea* , whose network of associated terms contains the terms “acne rosacea,” “disorders rosacea,” “rosacea,” and “rosacea acne.”

Because the concept *acne rosacea* incorporates at least one term containing the text string “acne,” its entire network of terms automatically became candidates for the *acne* lexicon. This inclusiveness raises the question of whether “rosacea,” “acne rosacea,” “disorders rosacea,” etc denote *acne*. To solve this problem, a physician on the research team vetted the candidate terms. For each disease, she dropped candidates that did not denote the disease, ensuring that only medically appropriate terms were admitted into any disease lexicon.

#### Structural

We produced a list of text strings for each disease that we could use to search the Twitter corpus for mentions of that disease. To achieve this goal we took into account two realities. First, many CHV term elements use constructions that are uncommon in natural language (eg, “fever hay” as in *nasal allergies/hay fever* ), “attack heart,” “attacks hearts,” or “attacking heart” as in *heart attack* , and “pain, back, radiating” as in *back pain* ). Second, during execution of searches within the Twitter corpus, only the shortest element of a search phrase is required; if a compound search phrase contains a shorter search phrase, the longer one is implied by the shorter (eg, “asthma” retrieves *asthma* , *allergic asthma* , *pollen asthma* , etc; “diabetes” retrieves *diabetes mellitus* , *insulin-dependent diabetes* , *diabetes screening* , etc).

Because of these two facts, we were able to significantly shorten the candidate disease-term lists that were produced by the semiautomated CHV search procedure. All reverse-order candidates (eg, “fever hay”) and compound candidates (eg, “allergic asthma”) were dropped.

After these three vetting procedures, the 24 disease lexica contained a combined 488 disease terms (see Multimedia Appendix 1).

### Manual Tweet Appraisal

We determined how often each of the 488 disease terms referred to its associated disease when included in a tweet. This began with a basic count for each disease term of the number of tweets in the corpus in which the term appears (before any language ambiguity corrections were applied). This is a disease term’s raw tweet count. Note that we allow a single tweet to be counted two times if it contains multiple disease terms (regardless of whether the two terms refer to the same or a different disease). Throughout this study, we consider random instances of disease terms as they appear in tweets, without consideration for other terms that co-occur with them.

We then performed manual appraisal. For each disease term, we randomly selected 30 tweets containing the term from our tweet corpus for manual analysis. This number was chosen to balance research needs and time constraints. Some disease terms occurred in 30 or fewer tweets in the tweet corpus. When this occurred, all available tweets were retrieved.

Two English-speaking research assistants independently read each tweet and made a simple appraisal, answering, “For each tweet, in your judgment does the disease term that flagged the tweet’s retrieval refer to a medical meaning of that term?” Each tweet required a Yes or No judgment, as shown in Table 1 . The two raters each compiled a complete collection of Yes/No judgments, held in secret from the other rater.

**Table 1:** Example of rating whether each tweet does or does not refer to a medical meaning of the selected term. Here the term is “heart attacks.”

Rater 1	Rater 2	Tweet
Yes	Yes	Visited a man who has had 2 heart attacks who feels privileged to be in circumstances that allow him to share his trust in God. #realdeal
Yes	No	Got room for 1 more? RT @pjones59: Sausage balls, heart attacks on a stick, dip, chips, wings and cheese, cream cheese/pickle/ham wraps
No	No	I still can't believe I saw Kris at work the other day. Talk about mini heart attacks. U_U

After these tweet-level appraisals were completed, we aggregated the scores at the disease-term level (independently for each rater’s collection of judgments). For each rater and each disease term (n=488), we calculated the percentage of tweets from the sample that were appraised as referring to a medical meaning. The Cohen’s kappa for inter-rater reliability was .77.

The disease-term percentages of the two raters were then averaged, resulting in a correction factor for each disease term. We multiplied this coefficient by the disease term’s raw tweet count ( *rcount* ), to arrive at an estimated disease-term *validated tweet count*( *vcount* ).

**Figure 1:**
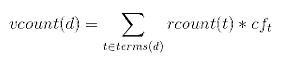
Equation for deriving a disease lexicon's correction factor.

Once this estimate was completed for each disease term in a disease lexicon, the disease term estimates were summed, producing our ultimate metric, a validated tweet count for each disease lexicon (Figure 1). The validated tweet count for a disease lexicon is the estimated number of tweets in our corpus that are a valid reference to the disease in question, that is, correcting for the ambiguity error present in the disease lexicon’s raw tweet count.

**Figure 2:**
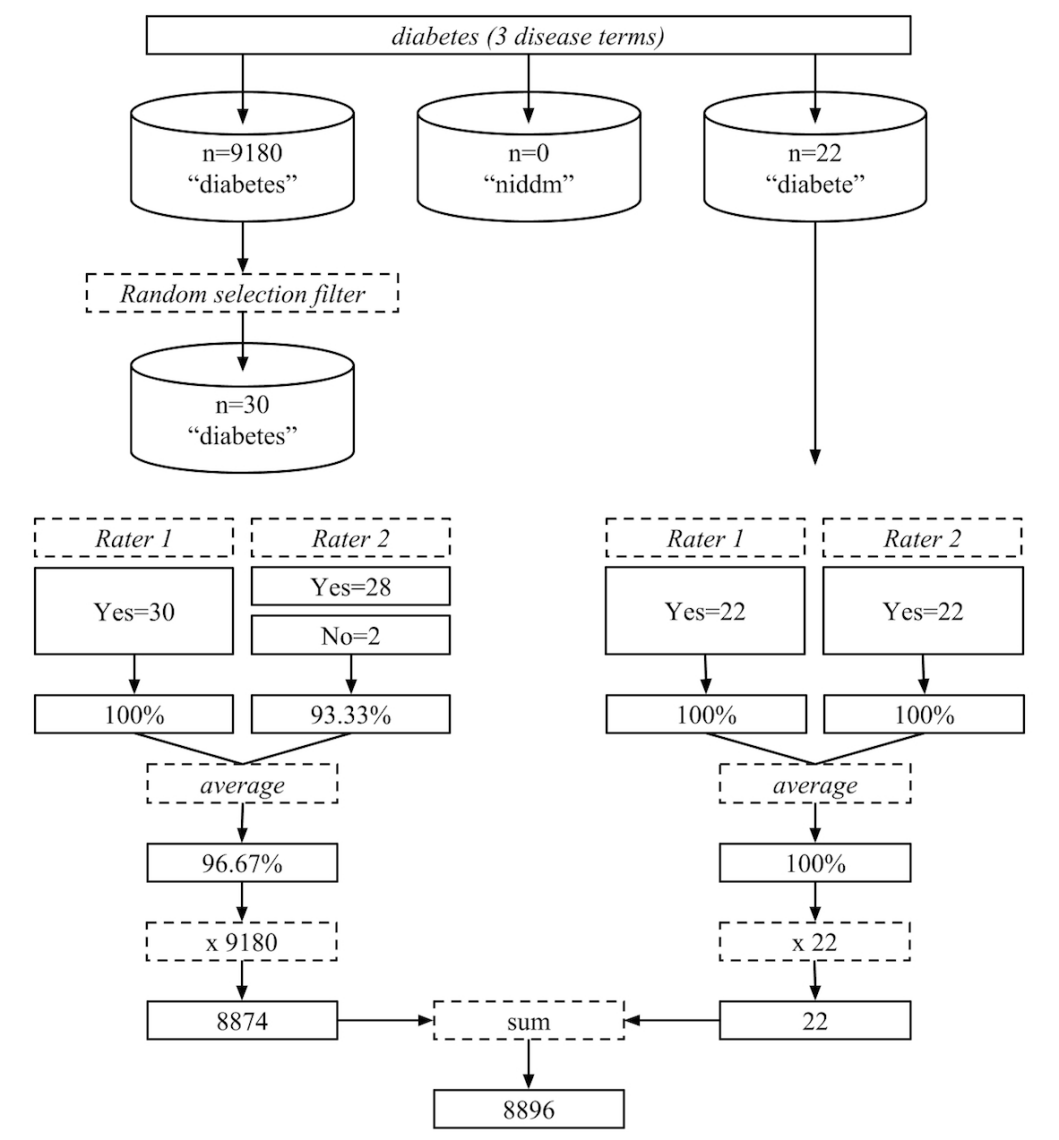
Disease terms from the diabetes lexicon that were subjected to manual appraisal. Each term receives appraisal on up to 30 instances. The term-level appraisals are then summed to reach the final lexicon-level diabetes-validated tweet count (8896).

As an example, manual appraisal for the *diabetes* disease lexicon (Figure 2) illustrates the evolution from a raw tweet count of 9202 to a validated tweet count of 8896.

## Results

### Preliminary Findings

Of the 2824 tweets containing disease terms that we manually reviewed, the averaged judgments of our 2 human raters indicate that 2276.5 (80.61%) actually referred to diseases, with validity rates that were highly variable across different diseases. For example, *stroke* terms rarely referred to the medical emergency (only 22% of the time, or 55/252), whereas *diabetes* terms almost always referred to the medical condition (98% of the time, or 102/104). Note that the percentages we report in Table 2 (14.89%, 3827/25,704, for stroke; 96.67%, 25,104/25,704, for diabetes) weight the manually derived percentages according to the term frequency in the Twitter corpus of the different terms that comprise a disease lexicon.

The raw tweet counts and validated tweet counts for the 24 diseases are compared in Table 2 , along with a correction factor (an adjustment according to the percentage of evaluated tweets that were judged as valid). Table 2 also includes disease-prevalence data (for both the general US population and among US Twitter users), which come directly from Experian’s Simmons National Consumer Study. We noted high levels of heterogeneity for all five measurements across diseases. This probably reflects the heterogeneity among the diseases themselves: among them are acute viral infections (eg, *flu* ), general maladies (eg, *backache* , *nasal allergies/hay fever* ), chronic disorders (eg, *arthritis* , *osteoporosis* ), test measures (eg, *high cholesterol* , *hypertension/high blood pressure* ), medical emergencies (eg, *heart attack* , *stroke* ), and psychological disorders (eg, *depression* , *ADD/ADHD* ). Some of the diseases are transitory (eg, *urinary tract infection* ) and others are long term (eg, *diabetes* ). Some are causes of mortality (eg, *cancer* , *congestive heart failure* ), whereas others are relatively superficial (eg, *acne* ). Given such variety, it is no surprise to see a wide range of values for tweet counts, correction factor, and prevalence across the list of diseases.

**Table 2:** Raw and validated tweet counts, correction factor, and US and Twitter disease prevalence for each disease.

Disease	Raw tweet count	Validated tweet count	Correction factor^a^	Prev US (millions)^b,d^	Prev US Twitter (millions)^c,d^
Acid reflux disease/gastroesophageal reflux disease	743	631	84.98	32.4	2.40
Acne	6936	6027	86.89	11.2	2.00
Attention deficit disorder/attention deficit hyperactivity disorder	2794	2660	95.19	4.9	0.90
Arthritis	2524	2522	99.92	34.4	1.30
Asthma	3952	3754	95.00	12.4	1.00
Backache	3035	3028	99.77	42.0	2.60
Cancer	110,760	63,647	57.46	5.0	0.46
Congestive heart failure	928	313	33.76	—	—
Heart disease	2741	2410	87.91	—	—
Congestive heart failure/heart disease^e^	3669	2723	74.21	5.9	0.46
Chronic obstructive pulmonary disease	226	188	83.37	5.5	0.86
Depression	14,294	10,459	73.17	18.7	2.20
Diabetes	9202	8896	96.67	20.8	1.20
Flu	10,139	8810	86.90	17.2	1.80
Genital herpes	76	66	86.84	1.8	0.33
Heart attack	15,027	2311	15.38	—	—
Stroke	12,852	1914	14.89	—	—
Heart attack/stroke^f^	27,879	4225	15.15	3.0	0.11
High cholesterol	225	218	96.67	37.9	1.70
Human papilloma virus	636	545	85.73	1.5	0.12
Hypertension/high blood pressure	1630	1491	91.49	43.5	1.50
Migraine headache	5958	5615	94.24	16.4	1.80
Nasal allergies/hay fever	481	473	98.27	18.2	1.30
Osteoporosis	316	306	96.68	6.0	0.13
Stomach ulcers	80	73	91.25	3.3	0.03
Urinary tract infection	880	479	54.40	10.0	1.00

^a^Correction factor is the percentage of tweets that were appraised as valid.

^b^Prev US (millions) represents a disease’s prevalence in the US.

^c^Prev US Twitter (millions) represents a disease’s prevalence among US Twitter users.

^d^The source for both Prev US (millions) and Prev US Twitter (millions) is the Experian Simmons National Consumer Study.

^e^In the Experian dataset, congestive heart failure and heart disease are collapsed into a single data point. We mined Twitter for these diseases separately, and we applied our evaluation method to tweets containing disease terms for each one separately. However, because Experian was our source for prevalence statistics, we can only report on the prevalence of these two diseases in a combined state.

^f^Note “e” is true for the diseases heart attack and stroke.

#### Statistical Analysis

We determined the Spearman correlation coefficients (all *P* <.001) between both raw and validated tweet counts and disease prevalence among both the general US population and among US Twitter users (Table 3). Correcting just for Twitter use more than doubles the correlation between tweet count and prevalence (from .113 to .258). Correcting only for word ambiguity has a similar but slightly smaller effect (.208). Correcting for both more than triples the baseline correlation (.366).

**Table 3:** Spearman correlation coefficients between both raw and validated tweet counts and US population and Twitter-user disease prevalence (all *P* <.001).

	Prevalence	
	US population	US Twitter users
Raw tweet count	.113	.258
Validated tweet count	.208	.366

## Discussion

### Overview

The correlation improvements we found due to ambiguity correction may seem unsurprising. However, the improvements due to demographic correction are less straightforward, particularly because no effort was made to restrict our tweet analysis to first-person self-report mentions of diseases. It is easy to assume that there must be a causal connection between disease prevalence and disease mentions. Indeed, we interpret an increased correlation due to demographic correction as supporting this assumption: it means the signal we measure (ie, disease mentions on Twitter) demonstrates positive correspondence to a plausible source of that signal (ie, disease sufferers who use Twitter). However, we find that for certain individual diseases, disease prevalence and disease mentions are wildly out of sync. For the time being just what causes someone to tweet (or not tweet) about a disease remains an open question, particularly because many people mentioning the disease are not suffering from it. In any case, methods utilizing social media to estimate disease prevalence do not need to explain a causal connection. They only demand that social media reliably captures the variance of disease prevalence. We have shown that such measurement can be improved by adjusting for demographic differences between disease sufferers and Twitter users.

### Bias Correction

We found that naïvely counting mentions of disease terms in tweets produces results that are biased (in terms of correlation with known disease-prevalence statistics) due to both demographic pattern of Twitter users and the ambiguity of natural language. These biases can be at least partially corrected, resulting in a threefold increase in the correlation between counts of disease terms in tweets and known prevalence statistics for the 24 diseases we studied.

The observation that the Twitter population is a biased sample of the United States is relatively easily corrected using standard stratified sampling methods, given the known demographics of the Twitter population. We identified this using data from an Experian survey, but other studies of Twitter demographics could also be used. We demonstrated that the demographic corrections roughly doubled the correlation between disease mentions and disease prevalence.

### Types of Ambiguity

The intrinsic ambiguity of language requires more work to correct. We observed that language ambiguity varies significantly across diseases. The fraction of mentions of a disease term that actually refer to the disease ranged from highly specific terms such as arthritis (99.92%, or 5044/5048) to less specific terms such as stroke (14.89%, or 3827/25,704). This language ambiguity takes 2 major forms.

The first is “lexical ambiguity.” Some diseases such as arthritis, diabetes, and high cholesterol are in practice referred to by terms that almost always refer to their associated disease concepts. In our analysis, tweeters rarely used words from the arthritis lexicon to refer to anything other than the disease “arthritis.” There are, however, a number of disease terms that are often used to refer to concepts that are not diseases (or not the intended diseases). Frequently occurring example words include “cancer” (the astrological sign), “depression,” “stroke” (nonmedical usages and also heat stroke), and “flu” (as in stomach flu, versus “influenza”). Abbreviations are particularly ambiguous. For example, “copd” (ie, chronic obstructive pulmonary disease) is a popular variant spelling of “copped” (as in the verb “took”); “uti” ( *urinary tract infection* ), “hpv” ( *human papillomavirus* ), and “zit” ( *acne* ) show up in Internet addresses (particularly in short links using URL redirection); and “CHF” ( *congestive heart failure* ) is an abbreviation for the Swiss Franc. Or conversely, “Gerd” is a masculine first name that coincides with an abbreviation for the disease gastroesophageal reflux disease (part of the *acid reflux disease/GERD* lexicon). Lexical ambiguity also arises from metaphorical and slang usages of disease terms. “Heart attack” and “heart failure” are used to mean surprise and “ADHD” to mean distracted.

The second type of ambiguity could be considered “disease ambiguity.” Some of the 24 diseases included in this study are less clearly delineated than others. One aspect of this problem is intensity. Is it medical depression if a Twitter user reports being depressed about her favorite sports team losing a game? What if she ends a seemingly grave tweet with “LOL?” A second aspect of disease ambiguity is specificity or accuracy. Some Twitter users may use the word migraine for other types of headache or say hay fever when actually they are allergic to cats. A third aspect of disease ambiguity is complexity. A prime example is the range of cardiovascular diseases in this study (ie, *congestive heart failure* , *heart disease* , *heart attack* , *high cholesterol* , *hypertension* , possibly *stroke* ), whose inter-relations and exact boundaries are difficult or impossible to draw.

Both types of ambiguity can affect a disease’s validation coefficient. The first type, lexical ambiguity (eg, homographs or metaphorical word usage), is likely to affect the “back end” of the methodology, requiring corrections to the observed term counts (ie, using the method described in this paper). The second type, disease boundary ambiguity, presents problems on the “front end” and it makes tailoring the disease lexica difficult. This type of ambiguity raises the question of whether the potentially hierarchical relationship between congestive heart failure and heart disease, or the potentially causal relationship between high cholesterol and either heart attack or stroke, could or should somehow be encoded in the disease lexica.

In this research, we treated each disease lexicon as a stand-alone entity, and the effects of that decision are necessarily written into the results we derived. We can expect that diseases of a more “stand-alone” quality (ie, those that are relatively self-contained like *osteoporosis* , rather than part of a complex like *heart disease* ) will naturally be better represented by their respective disease lexica than are diseases that potentially harbor a complex relationship with other diseases. It is intuitive that mismatch between a disease’s representation on Twitter and its representation within its disease lexicon is essentially what causes the disease’s validation coefficient to drop below 100%.

### Correlation of Validated Tweet Count With Prevalence

Just as validity rates proved highly variable across diseases, the levels of Twitter discussion relative to disease prevalence also varied. Some diseases were discussed at levels outstripping their prevalence in the population, whereas others received little relative attention. The relationship between validated tweet count and US prevalence across diseases has a correlation of .208 (Table 3 , *P* <.001). To provide a more detailed picture of this relationship, we calculated validated tweet count for each disease as a function of prevalence. The following formula is used for this purpose: for each disease *d* , projected prevalence = (validated tweet count of *d* /sum of all validated tweet counts) × sum of all disease prevalence. This can be understood as the prevalence that validated tweet count (inaccurately) projects for each disease.

**Figure 3:**
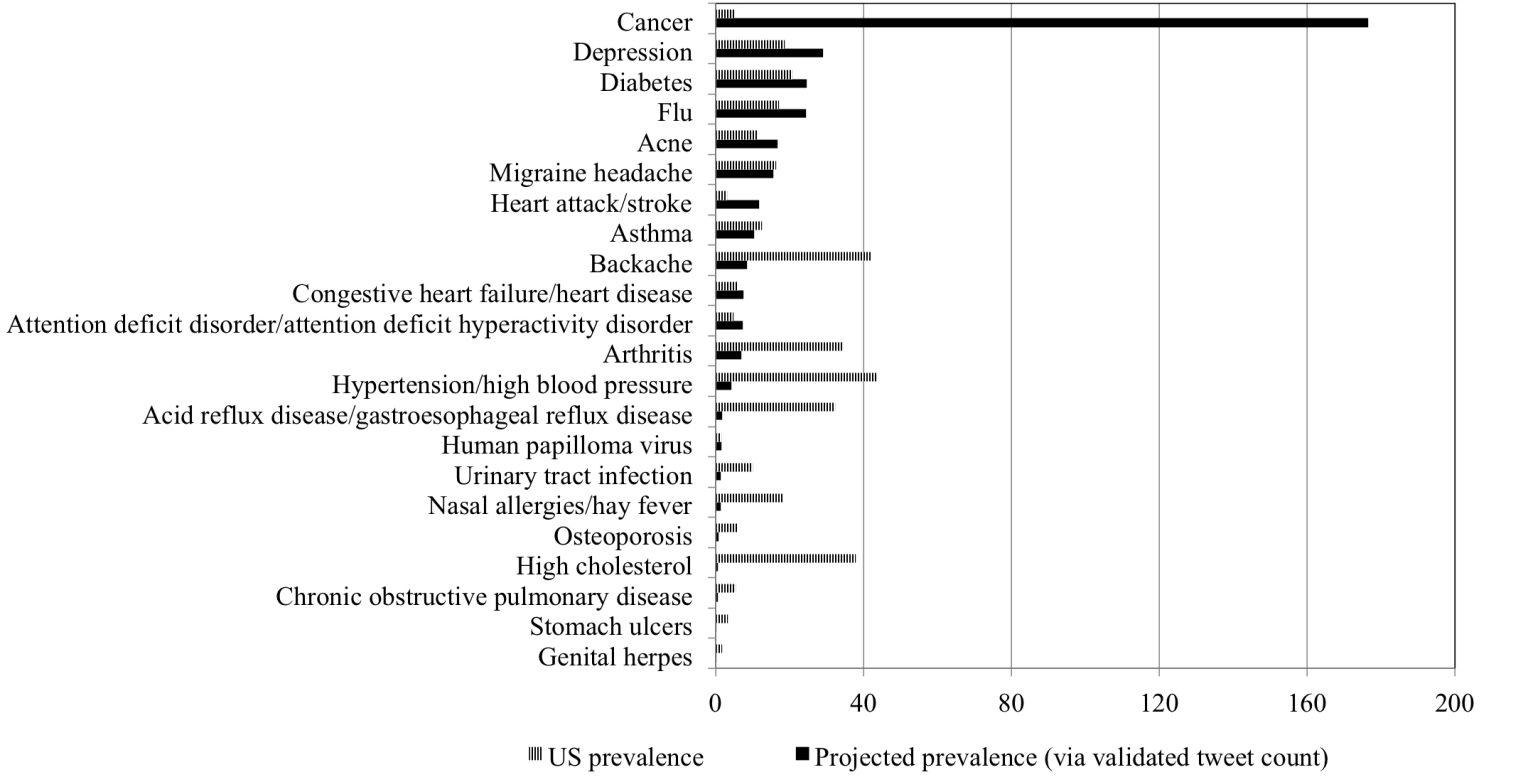
Projected prevalence (as a function of validated tweet count) versus actual US prevalence for 22 diseases, in millions (sorted by projected prevalence). Some diseases are “over-tweeted” (in particular, cancer), whereas others are “under-tweeted” (eg, backache and arthritis).

We compare this projected prevalence with actual prevalence in Figure 3 . The sum of prevalence across all diseases (351,939,580) is identical for both the projected and the actual cases, but the distributions are quite different. We see that *cancer* is a major outlier, “taking up” over 50% of projected prevalence (176,605,210), whereas it accounts for less than 2% of actual prevalence (5,031,120). Clearly, *cancer* receives far more attention than merely prevalence warrants. Projected prevalence is more than 35 times as great as actual prevalence. Conversely, *high cholesterol* is on the extreme end of underrepresentation. Projected prevalence (604,898) is only 1.60% of actual prevalence (37,861,070). These figures demonstrate that other unknown factors besides prevalence influence the amount of discussion a disease receives on Twitter.

One hypothesis is that Twitter demographics skew discussion levels upward for diseases that are of high concern to the population of users and downward for diseases that are of less concern. Given the generalization that Twitter users tend to be young, this could explain why *arthritis* seems to be drastically under-tweeted, and why *acne* and *ADD/ADHD* are over-tweeted. However, demographics alone cannot explain the extraordinary projected prevalence of *cancer*. Nor are they likely to explain the over-tweeting of *flu* and *diabetes*. We assume that demographics do influence these results (notice the over-projection of *acne* , a disease of youth, and the under-projection of *hypertension/high blood pressure* , a disease of aged, in Figure 3), but that multiple other factors also play roles. Likely candidates include the intensity and history of disease awareness and advocacy campaigns (see *cancer* , *diabetes* , and *human papilloma virus* ); and disease stigma or body-part stigma (see *genital herpes* and *urinary tract infection* ). Investigation into these and other possible factors is an area for future research.

### Limitations

It remains unclear precisely what is the nature of the relationship between disease discussion (on Twitter or even just in general) and disease prevalence. Twitter disease discussion is likely driven by many more factors than disease prevalence. People tweet about diseases for many reasons, and for the purposes of this paper, we do not attempt to disentangle such reasons. We do demonstrate, though, that Twitter disease mentions correlate with disease prevalence, and that this correlation improves after our demographic and word ambiguity corrections have been applied. This lesson can and should be incorporated into other research or tools that would seek to mine the language found on Twitter (or similar venues) for information about broader populations.

Despite our best efforts, the demographics of our Twitter corpus and of the Experian dataset do not entirely match. Most significantly, the Experian dataset includes disease-prevalence estimates for both English-speaking and Spanish-speaking US residents, whereas our tweet corpus was restricted to English language tweets. This research was conducted in English; future work should extend a similar analysis to other languages.

We did not account in this research for all possible variables that could influence the interplay of disease prevalence and tweets about diseases. Some of these missed variables are disease centric. For example, some diseases may actually be more “tweet-able” than others due to any number of disease factors, including intensity, duration, stigma, social salience, and so on, or possibly even due to formal considerations (is the disease easy or quick to spell?). A less tweet-able disease might be expected to have fewer associated tweets, outside of any prevalence-based influence on tweet counts.

We only account for mentions of diseases that specifically name a (properly spelled) disease in a tweet. On the one hand, relying on correct noun-form disease names that are sourced from a recognized health vocabulary such as the CHV helps push this study toward semiautomation, objectivity, and reproducibility. However, on the other hand, this decision leaves an unknown, but possibly large, quantity of disease-relevant tweets unmined, and so unaccounted for in our analysis. We miss mentions that are slang terms (eg, “diabeetus”) or are misspelled (eg, “ashtma,” “hi cholesterol”). On a strictly formal level, our current approach is tuned to precision at the expense of recall. Furthermore, people may discuss health concerns on Twitter by mentioning symptoms, sequelae, locations (such as a hospital), drugs, or treatments, etc. Our focus on disease names is unable to capture this broader domain of health-related tweets. Improving recall is left for future work.

Other missed variables are Twitter centric. It is well documented that Twitter does not reveal what sampling procedures are used in their APIs [27,28]. Therefore, it is unclear how representative the tweeters (whose tweets were captured for this study) are of the US population of Twitter users. This is unavoidable, and it is a shortcoming common to all research using Twitter APIs.

We also did not discriminate in this research between tweeters. A Twitter “user” may not be an individual person. Many health-related or even disease-related organizations mention diseases on Twitter. Factors related to such organizations (their quantity, their social media strategies, etc) may be relevant to counts of disease-naming tweets. Other researchers have addressed the problem of distinguishing tweets authored by health organizations [29], but this study did not make that distinction.

### Comparison With Prior Work

In the normal course of life or business, individuals and organizations generate vast amounts of text that can be mined. Much of it is shared or published online in one form or another, and these data are attractive to researchers, including those interested in epidemiology and public health.

Several “infodemiology” studies (eg, many in the long list cited in the “Introduction” section) correlate word use in Twitter with prevalence of a disease or medical condition. Beyond Twitter, Web search activity has also been used, for example to monitor Lyme disease [30] and dengue [31], as well as risk behaviors associated with dietary habits [32] and with suicide [33]. Blog posts have been used to predict influenza outbreaks [34]. Facebook has been used to predict “gross national happiness,” that is, well-being across the United States [35].

These studies primarily correlate word use in some medium (eg, Twitter or Google search) over some period (eg, day or week) and in some region (eg, US county or state) with a disease-prevalence level. Such correlational approaches rely on certain assumptions about the homogeneity of the populations they study, which often go unstated and so presumably untested and uncorrected. It is not clear whether demographic or word ambiguity biases are typically accounted for. We suppose that researchers implicitly assume that these factors will be handled automatically by the statistical regression methods they use. If demographic and ambiguity biases are constant over time and space, this will be true. However, if populations vary in their usage of the target medium (eg, Twitter), prediction accuracy can vary, and this variation may be significant.

Google Flu Trends is perhaps the “poster child” for the correlational approach to prediction. It is a widely cited online tool that uses statistical correlations between a broad set of Google search terms and historical flu levels to predict regional changes in US flu levels [36]. Google Flu Trends was initially highly accurate. However, it has also been used as a case study of how “big data” predictions can go awry when the statistical patterns upon which they are based are descriptively inaccurate (either from the start or due to drift over time) [37,38], with claims that at one point predictions became exaggerated by nearly a factor of 2 [39].

A limited number of studies have emphasized concerns about validity in social media analyses [40]. There has also been some work on selecting “high-quality” disease-related tweets, mostly achieving high specificity at the cost of poor sensitivity. For example, [41] uses regular expressions and machine-learning methods to filter out all but first-person self-report tweets. We strive for higher coverage, including all “real” mentions of a disease, and then we seek out previously established data (ie, disease prevalence) against which we validate our findings. In a previous study [42], researchers identified known sick persons, then study their Twitter data to characterize a kind identifying fingerprint for Twitter users who have the flu (utilizing their tweets and their Twitter profile metadata). They then use that model to “diagnose” individual Twitter users with influenza, an approach that the authors imply could be directed toward population-level disease surveillance.

In the near term, we think that the major use of social media for public health may be to understand attitudes toward health, disease, and treatment. Effective public policy depends on subjective inquiries into what people know and care about. Why do they seek or avoid treatment? How do they reveal disease status? What risk behaviors do they shrug off? Predictions about a phenomenon that one can measure, such as disease prevalence, may have limited utility, especially if the measurements are timely and accurate. Although traditional ground truth measurements have been questioned [43], the Centers for Disease Control and Prevention flu estimates appear to be better than Google Flu Trends estimates [44]. Nevertheless, online disease detection and prediction is a rapidly growing research area, and as work continues in this field, our collective ability to make these types of estimates will likely increase.

### Conclusions

Several types of research using social media to study public health will benefit from corrections for demographic variation and language ambiguity of the type that are outlined in this paper. Social media datasets are biased convenience samples, and word ambiguity is endemic. Nevertheless, social media provide a relatively cheap way to monitor countless domains, including public health and attitudes toward health and health care.

In this study, we began with a large, “poor-quality,” nonrandom dataset (ie, Twitter), and compared it with a small, “high-quality,” random (achieved via poststratification) dataset (ie, the Simmons National Consumer Study from Experian). We filtered the Twitter dataset so that its demographics would match that of the Experian dataset. We then performed both naïve and ambiguity-corrected counts of Twitter disease mentions. Finally, we compared both of these counts with prevalence data found in the Experian survey. We found that the corrected Twitter counts correlated much more strongly than the naïve Twitter counts with the “high-quality” Experian data.

We think that this demonstrates both the need and the capacity for other studies using nonrandom convenience samples (eg, social media data or Google queries) to take demographic and word ambiguity factors explicitly into account, for example, using our method or other related or novel methods.

## Appendix

Multimedia Appendix 1 This study focuses on a list of 24 diseases. Each of these diseases is represented by a disease lexicon composed of one or more disease terms. There are 24 lexica, comprising 488 disease terms. The first row in this appendix holds lexica names, subsequent rows hold disease terms. Each column represents a different disease.

## Abbreviations

ADD/ADHD: attention deficit disorder/attention deficit hyperactivity disorder
API: Application Programming Interface
CHV: Consumer Health Vocabulary
UMLS: Unified Medical Language System
